# Modeling solutions to Tanzania's physician workforce challenge

**DOI:** 10.3402/gha.v9.31597

**Published:** 2016-06-27

**Authors:** Alex J. Goodell, James G. Kahn, Sidney S. Ndeki, Eliangiringa Kaale, Ephata E. Kaaya, Sarah B. J. Macfarlane

**Affiliations:** 1 School of Medicine, University of California San Francisco, San Francisco, CA, USA; 2 Philip R Lee Institute for Health Policy Studies, University of California San Francisco, San Francisco, CA, USA; 3 Independent consultant, PRAXIS, Tanzania; 4 School of Pharmacy, Muhimbili University of Health and Allied Sciences, Dar es Salaam, Tanzania; 5 School of Medicine, Muhimbili University of Health and Allied Sciences, Dar es Salaam, Tanzania; 6 Global Health Sciences, University of California, San Francisco, CA, USA

**Keywords:** workforce, modeling, doctor shortage, Tanzania

## Abstract

**Background:**

There is a great need for physicians in Tanzania. In 2012, there were approximately 0.31 physicians per 10,000 individuals nationwide, with a lower ratio in the rural areas, where the majority of the population resides. In response, universities across Tanzania have greatly increased the enrollment of medical students. Yet evidence suggests high attrition of medical graduates to other professions and emigration from rural areas where they are most needed.

**Objective:**

To estimate the future number of physicians practicing in Tanzania and the potential impact of interventions to improve retention, we built a model that tracks medical students from enrollment through clinical practice, from 1990 to 2025.

**Design:**

We designed a Markov process with 92 potential states capturing the movement of 25,000 medical students and physicians from medical training through employment. Work possibilities included clinical practice (divided into rural or urban, public or private), non-clinical work, and emigration. We populated and calibrated the model using a national 2005/2006 physician mapping survey, as well as graduation records, graduate tracking surveys, and other available data.

**Results:**

The model projects massive losses to clinical practice between 2016 and 2025, especially in rural areas. Approximately 56% of all medical school students enrolled between 2011 and 2020 will not be practicing medicine in Tanzania in 2025. Even with these losses, the model forecasts an increase in the physician-to-population ratio to 1.4 per 10,000 by 2025. Increasing the absorption of recent graduates into the public sector and/or developing a rural training track would ameliorate physician attrition in the most underserved areas.

**Conclusions:**

Tanzania is making significant investments in the training of physicians. Without linking these doctors to employment and ensuring their retention, the majority of this investment in medical education will be jeopardized.

## Introduction

To provide universal health care by 2030, the World Health Organization (WHO) estimates that the world will need an additional 10.1 million health care workers (1). Africa is the continent with the highest need, with a predicted deficiency of 3.7 million health workers (1). In particular, there is a shortage of physicians; in 2008, Scheffler et al. estimated that an additional 120,000 to 230,000 doctors were needed by 2015 to meet the health needs of Africans (2, 3). Governments and private institutions make large investments to train these highly-qualified individuals (4). However, doctors are more likely than other health care workers to migrate abroad, stay in already well-served urban areas, or enter non-clinical professions such as non-profit management (5, 6). To protect their investments in medical education, governments must examine how best to encourage doctors to serve national needs (7).

The Tanzania Development Vision 2025 commits the nation to providing ‘access to quality primary health care for all’ (8). To fulfill this vision, the government is working to increase the health workforce, including expanding the number of doctors from the most recent estimate of 0.31 doctors per 10,000 population to meet the demands of the population (9). In line with this effort, Tanzanian universities are expanding their medical student intakes dramatically. Whereas in 1991 the one medical school in Tanzania admitted 55 students, in 2015 the Tanzanian Commission for Universities approved 11 medical schools to admit a total of 1,580 students per year (10). It is vital that these students become quality physicians who practice clinical medicine where they are most needed in Tanzania.

Leon et al. reported that, on entering medical school, students in Tanzania in 2005 were more interested in good salaries and social status than in practicing medicine and that, on graduation, only one-third reported being more motivated towards medicine than when they entered school (11). Upon graduation, many medical graduates cannot find employment. For example, in 2010 the government provided only 280 immediate postings to 632 medical school graduates (12). There is little evidence as to where the other new doctors went (13), although some may have left the country or obtained non-clinical positions (13–15).

Rural areas are severely underserved by health workers. In 2006, only 20% of doctors practiced in rural areas where 73% of Tanzania's population live (5). Young doctors resist rural placement: according to Sikika, 26% of Tanzanian medical graduates did not report at their rural posting in 2009 (16). In another study, 48.5% of medical students said they would reject a posting to a rural area, giving adverse working conditions as the primary reason (17).

The Tanzanian government, universities, and national accreditation body need to revisit the investments being made in physician education. This includes understanding what happens to doctors when they graduate – whether they work as clinicians in Tanzania and where they are most needed in rural areas. Any assessment should also include an evaluation of potential strategies to ameliorate the loss of trained physicians from the health workforce.

Mathematical simulation models of human resources are useful tools in long-term strategic planning (7). As part of a collaboration between the Muhimbili University of Health and Allied Sciences (MUHAS) and the University of California San Francisco (UCSF), we developed the Increasing Clinically Active Doctors (ICAD) tool, an interactive, mathematical model that predicts the distribution and flow of doctors through the educational and health system. We designed the tool to assist policy makers to look at options for providing equitable access to physicians throughout the country.

We describe how the model tracks cohorts of medical students through career pathways in Tanzania and identifies losses to the system. We make projections about the numbers of clinically active doctors up to 2025 and about the number working in rural areas. We demonstrate huge losses to the current national investments in physician education and highlight the need for reforms to retain and incentivize physicians to work where they are most needed. We conclude by highlighting the relevance of our findings for other countries that are expanding their physician education programs.

## Methods

### Career pathways for doctors in Tanzania

**Fig. 1:**
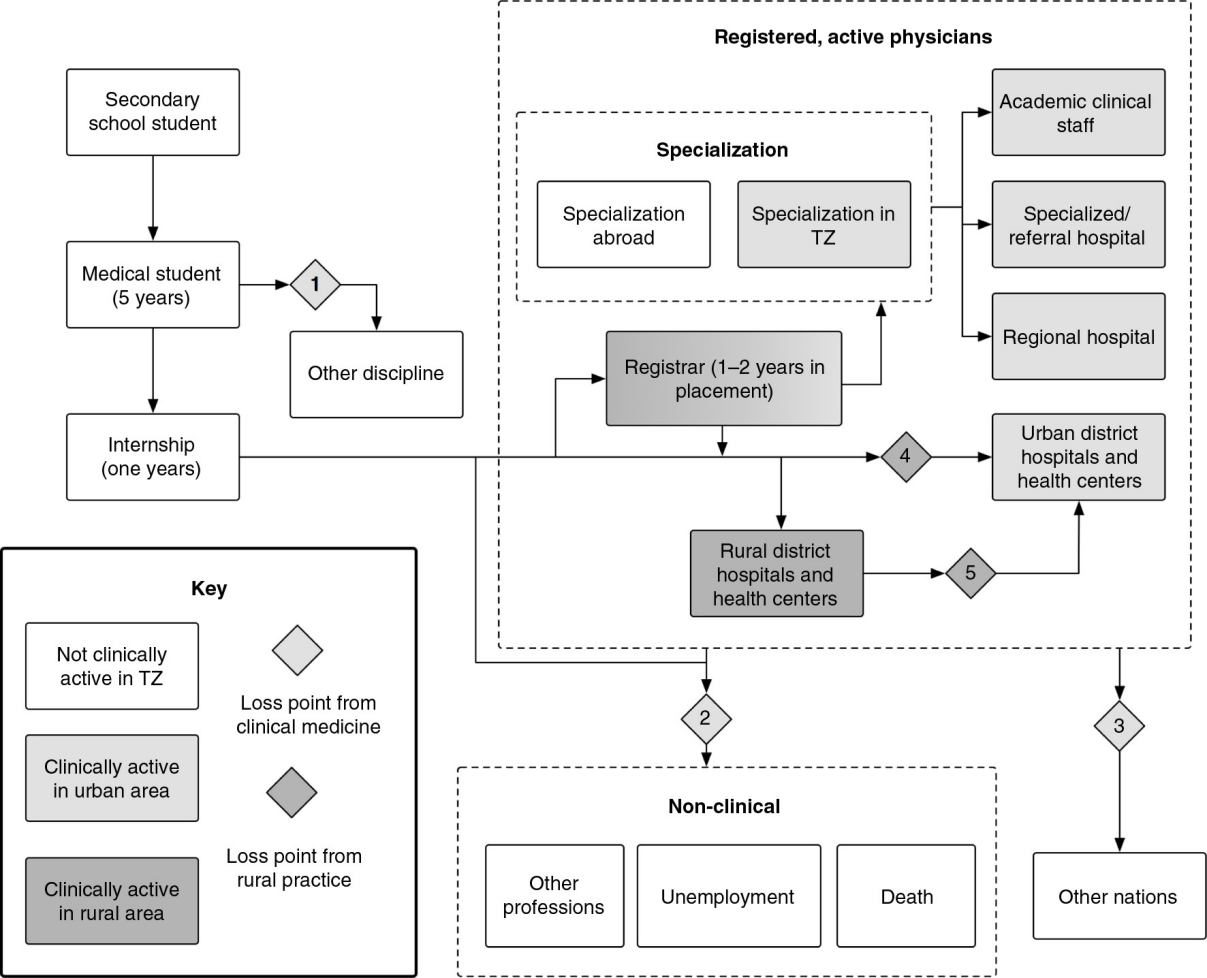
Pathways through medical education and graduate employment in Tanzania.

After discussion with Tanzanian stakeholders and medical education experts, we identified the major career pathways for doctors in Tanzania. We focused on when doctors enter or leave clinical practice and enter or leave rural areas (Fig. 1).

Secondary school students qualify for medical school on the basis of A-level exams. After a 5-year medical training program, most graduates take up a 1-year internship at a national/referral hospital or regional/municipal hospital, where they learn under the guidance of registered medical doctors. Upon completion of that year, they register with the Tanganyika Medical Council. Most continue to public service positions as registrars for about 2 years. Others bypass the registrar period and directly enter general practice in rural or urban areas. After at least 2 years of clinical practice, some individuals will enter specialized training programs, after which they are posted to regional, specialized/referral, or academic hospitals, often in urban areas.

We identified major loss points (LPs) in the system whereby clinically active doctors are ‘lost’ from the most-needed areas by transitioning to non-clinical positions or from rural areas to urban areas (Fig. 1). Some medical students change disciplines while at university or do not complete their course (LP1); some decide not to pursue clinical careers after graduation; and others change careers after practicing, become unemployed, or die (LP2). Some doctors leave to work abroad (LP3). Most clinically active doctors choose not to practice in rural areas (LP4) and some later shift from rural to urban areas (LP5).

### Model structure

We developed a mathematical model in Excel version 15.17 using a Markov state process (18, 19) with 92 potential states to predict the movement of doctors through the pathways described in Fig. 1. We based the input data on available data (such as past medical school enrollment, reported migration rates, tracking surveys of graduates) and then calibrated the model by estimating and adjusting variables until the resulting figures represented the majority of our evidence. In this way, we created a model that dynamically represented the situation described in a national facility mapping survey undertaken in 2005/2006 (5). Table 1 provides the model parameters. The model utilized constant transition probabilities between the various states, except for the number of individuals choosing to work in the public sector, which was modeled as a function of the number of available positions.

**Table 1:** Input parameters to the model

Input parameter	Best estimate used in model	Source
Total medical school graduates (selected years)		
1990	35	Number of admissions approved by the Tanzania Commission for
1995	35	Universities in 2015 (10), MUHAS records of graduates (21),
2000	49	and (15).
2005	170	
2010	400	
2015	675	
2020	1,422^a^	
Number of public positions available to graduates each year	280	Number available in Tanzania in 2010 (12).
Percent of graduating physicians who practice entirely in the public sector	45%	Varies in the model depending on the number of public positions available and number of graduates seeking clinical practice.
Percent of students who do not graduate (LP1)	10%	No data for Tanzania; estimate based on rates from the United Kingdom (25).
Percent of graduates who choose not to practice clinically (LP2)	20%	Used by Bryan et al. in their model of Tanzanian human resources for health (26). Leon et al. report 21% of Tanzanian students plan on studying for a master's in public health (11).
Percent of clinically active physicians who leave to NGO positions per year (LP2)	2.5%	Assumed and calibrated in the model to match graduation data and 2006 mapping survey (5).
Percent of clinically active physicians who enter non-health-care positions per year (LP2)	2%	Assumed and calibrated in the model to match graduation data, 2006 mapping survey (5), and Sikika tracking survey (45).
Percent of clinically active physicians who leave Tanzania per year (LP3)	1.6%	Assumed and calibrated in the model to match (2, 45, 47).
Annual mortality rate	0.9%	World Development Indicators for Tanzania, 2014 (27).
Percent of graduates who do not take up positions in rural areas (LP4)	26%	Estimated for Tanzania in 2010 by Sikika (16).
Percent of employed graduates who leave rural areas per year (LP5)	3.3%	Assumed and calibrated to match graduation data and the 2005/2006 Tanzania Service Availability Mapping survey (5).
Population living in urban areas	27%	Estimated by the 2005/2006 Tanzania Service Availability Mapping survey (5).
Population living in rural areas	73%	Estimated by the 2005–2006 Tanzania Service Availability Mapping survey (5).
Population growth rate	3%	World Development Indicators for Tanzania, 2014 (27).

a Assuming 90% of enrollees graduate. MUHAS, Muhimbili University of Health and Allied Sciences; LP, loss point.

We created a class of medical student admissions for each year starting in 1990 and tracked their movements using a yearly time-step through graduation and until 2025, the end point of the Tanzanian Development Vision 2025 (8). Until 2001, MUHAS was the only accredited university producing medical doctors (20). To create a retrospective cohort, we used MUHAS graduation data from 1990 to 2000 (21), data for 2001–2010 graduates from all schools reported by Sirili et al. (15), and approved admissions information from 2011 to 2015 from the Tanzania Commission for Universities (10, 22–24). For the prospective cohort, we assumed a constant number of admissions equal to the number approved by the Tanzania Commission for Universities for the 2015/2016 academic year (10), a figure that may be higher than the actual number recruited. Records were insufficient to estimate the number of students who dropped out of medicine by changing disciplines or not graduating; we used an estimate of 10% of all admissions suggested by a study from the United Kingdom (25). We assumed that 20% of graduates did not enter the clinical workforce after graduation, as suggested by Bryan et al. (26).

We classified graduate doctors in each time-step by their state in the system laid out in Fig. 1, as well as by whether they were clinically active and whether they worked in urban or rural areas. A doctor could become clinically inactive by undertaking a master's degree in public health, taking a position with an NGO, taking an administrative position, choosing another profession, becoming unemployed, or dying. We classified the remaining doctors as clinically active, employed in either the public or private sector. We constrained the number employed from graduation by the available government positions, which were set at 280, the figure available in 2010 (12). We assumed an average 7% annual growth in private sector employment, consistent with gross domestic product growth (27).

We categorized as rural all districts outside of major cities without a regional hospital (73% of the population) and all other areas as urban. The 2005/2006 national facility mapping exercise confirmed that the areas we classified as urban were the most heavily populated with doctors (1.15 per 10,000 population), versus rural areas at 0.1 per 10,000 (5).

The Tanzanian government has not specified how many physicians it needs in order to fulfill its Development Vision 2025 and meet the health needs of the population. An often-cited physician-to-population recommendation comes from the 1993 World Development Report, suggesting a level between one and two physicians per 10,000 population as a minimum (28), compared to a global average of 14 per 10,000 in 2012 (9). A detailed model of the health needs of Tanzania, a nation with a high burden of disease, estimated a minimum requirement of 1.5 physicians per 10,000 population by 2015, once non-physician medical and clinical officers (COs) were excluded (29).

## Results

### Projected numbers of doctors

**Fig. 2:**
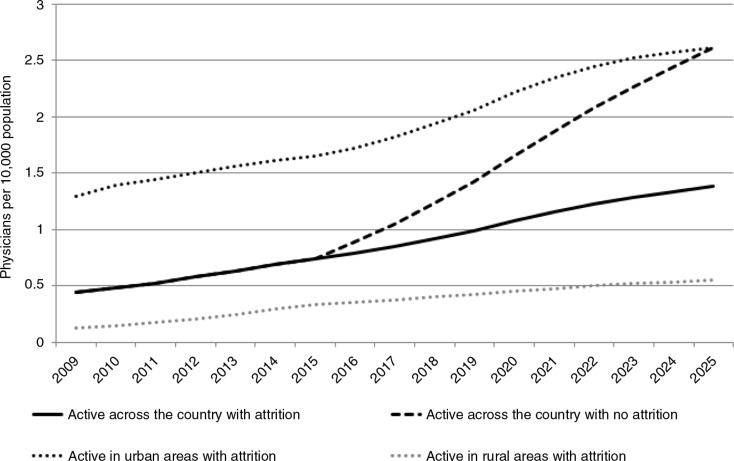
Projected density of clinically active physicians in urban areas, rural areas, and overall, 2009–2025, with and without attrition in 2015 graduates onward.

Figure 2 shows the career trajectories of graduating students, assuming that admission rates remain constant at 1,580 a year from 2015. The model predicts that in 2025, with no attrition, there would be approximately 2.6 practicing doctors per 10,000 population in Tanzania. That is, if all students admitted actually graduate and stay in clinical practice, there will be an eightfold increase in the physician-to-population ratio from 2012 levels. However, the model predicts a steady loss of doctors to the health system, with about 44% of all Tanzanian-trained doctors practicing in Tanzania in 2025 and thus a ratio of 1.4 practicing doctors per 10,000 population (Fig. 2). Further, with these trends and physician preferences to work in urban areas, the ratio of practicing doctors to population in 2025 would be 0.55 per 10,000 in rural areas versus 2.6 per 10,000 in urban areas.

### Losses from clinical practice

**Fig. 3:**
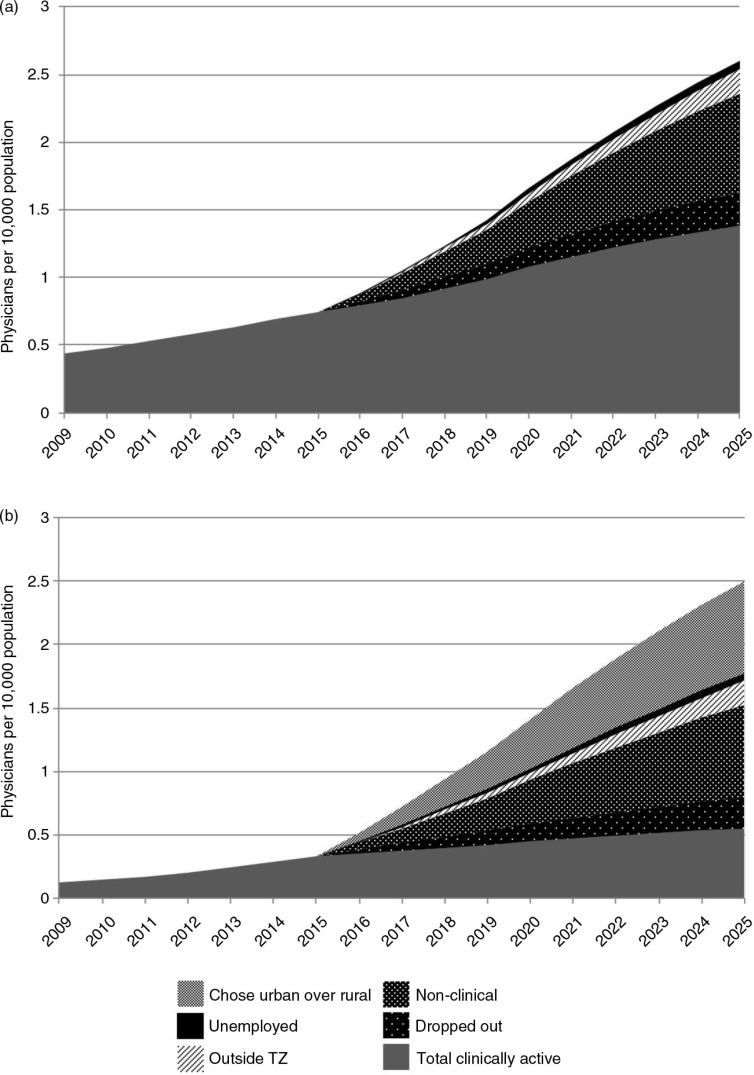
(a) Projected density of clinically active physicians and those lost to clinical practice, 2009–2025; (b) projected density of clinically active physicians in rural areas and those lost to rural clinical practice, 2009–2025.

Figure 3a demonstrates the distribution of losses of doctors to clinical practice from 2016 onwards, with reasons for attrition. By 2025, 56% of all medical school students enrolled between 2011 and 2020 will not be practicing medicine in Tanzania. The greatest and escalating loss is not through emigration but through doctors leaving the workforce or taking up non-clinical positions (more than 80% of the total loss). The figure also shows the impact of students not graduating (at the assumed rate of 10%).

### Losses from rural areas

Figure 3b shows the distribution of doctor losses from rural areas from 2016 onwards. Beyond the losses shown in Fig. 3, the graph indicates the loss to rural areas of doctors not accepting rural posts or leaving them to return to urban areas. This represents 37% of all rural losses.

### Impact of interventions

We developed ICAD to enable policy makers to evaluate interventions to address the identified key LPs. ICAD allows the user to specify an intervention and hypothesize the impact on the LPs. We illustrate the model's predictions for two interventions.

#### Increase the number of public sector clinical positions available

Many medical graduates are unable to find government employment. In 2010, the Tanzanian government provided only 280 immediate post-graduate postings, though there were over 632 medical school graduates (12). This situation has persisted since 2010 (15). This is particularly problematic for rural areas, as approximately 83% of clinically active doctors in rural areas worked in public facilities, according to the 2005/2006 mapping project (5). In order to employ the increasing numbers of graduating medical students who expect to enter the public sector, the government needs to scale up the number of available positions. If, for example, the government were to offer 800 positions for its graduates (just over half of the matriculating students), we estimate that the nation would reach 1.68 doctors per 10,000 population by 2025. Smaller increments in the number of students absorbed would still have a notable effect (Table 2).

**Table 2:** Predicted numbers of clinically active physicians resulting from increasing the number of public positions available to recent graduates

Number of public positions offered	Number of clinically active physicians per 10,000 population, nationwide, by 2025
280	1.33
400	1.41
500	1.48
600	1.55
700	1.61
800	1.68

#### Fellowship for students from rural areas to increase the number of clinically active doctors taking up rural positions

Studies suggest that students with parents in rural areas and those expressing a desire to ‘help the poor’ are more likely to accept rural postings (11, 30). Students from rural areas in South Africa and Washington State, USA, were 300–800% more likely to accept a rural posting after undertaking a rural training track (31, 32). We used the model to explore retention of doctors in rural areas through the development of a fellowship for rural students who plan on practicing in rural areas. Such fellowship programs require additional training and support during school. Assuming a program could accommodate 400 fellows and that 90% of those fellows enter rural areas with an annual attrition rate of 0.5% to urban centers (compared to the current estimate of 3.3%), the model projects a doubling of the number of doctors clinically active in rural areas by 2025. Scenarios using a different number of fellows or different assumptions about their acceptance of rural postings and rate of attrition still exhibit a favorable effect on rural physician-to-population ratios in 2025 (Table 3).

**Table 3:** Predicted number of clinically active physicians resulting from offering rural fellowships given the annual number of fellows and their characteristics

Number of fellows	Percent of fellows who choose to work in rural areas	Percent of fellows who leave rural areas to work in urban areas each year	Clinically active physicians per 10,000 population in rural areas by 2025
0	–	–	0.55
100	80	1	0.66
100	90	0.5	0.67
200	80	1	0.79
200	90	0.5	0.81
300	80	1	0.93
300	90	0.5	0.97
400	80	1	1.1
400	90	0.5	1.2

### Limitations

This model, like any other, is limited by uncertain input parameter values and structural assumptions. First, since we could not find appropriate data for many model parameters for doctor movement, we made assumptions and calibrated the model to reflect the doctor distribution in the 2005/2006 mapping survey. If the dynamics of doctors’ workforce choices are markedly different before or after the mapping survey, our assumptions are less realistic. Unfortunately, data on the current number of doctors are contradictory. The Ministry of Health and Social Welfare (MoHSW) published figures for the number of clinically active doctors that were considerably lower than our estimates: 1,481 medical doctors (including specialists) in 2012, only 200 more than the number reported by the 2006 mapping project (5, 33). For 2013, their estimate had risen to 2,194 (13). During 2012 and 2013, our model estimates 2,300 and 2,700 practicing doctors, respectively. Some of this discrepancy may be due to a 2012 doctors’ strike, whereby 300 physicians were dismissed (34); deficiencies in our model assumptions; or challenges encountered by the MoHSW's new human resources for health information system (35). Our model thus may overestimate the number of practicing physicians.

Second, the model does not capture the wide range in service availability, but simplifies the situation into ‘urban’, classified as those areas that were best served, and ‘rural’, classified as those areas that were least well served. This simplification may obscure significant heterogeneity in access in both urban and rural areas. It is possible that in areas with higher doctor densities, the nature of their employment (private practice, military hospitals) make them less accessible to the public. Conversely, it is possible that areas classified as rural bordered transportation systems that increased access to health facilities.

Third, the model does not account for mid-level medical providers, such as assistant-medical officers (AMOs) and COs. COs receive a 3-year post-secondary education in primary care and are meant to serve in rural postings. AMOs receive an additional 2 years of training after 3 years of experience as a CO and have training in emergency surgery and obstetrics. In 2006, there were slightly fewer medical doctors practicing than AMOs and over fivefold fewer than COs (5). These mid-level providers are essential components of the health care system and complement, but depend on, the availability of medical doctors (36). Future changes in the number of AMOs and COs may alter the required number of physicians.

Fourth, our model did not account for productivity. The predicted increases in the number of medical students in Tanzania may result in a lower quality of education, as there are limited clerkship and laboratory spots as well as strained teacher-to-student ratios (37, 38). With the large number of new students graduating, the majority of doctors in Tanzania over the next 10 years will be relatively inexperienced junior doctors, who, combined with insufficient clinical experience during medical school, may not be able to meet the health care demands of the population. In addition, clinics are often challenged by lack of equipment, poor supervision, and absenteeism (39, 40). Because our model did not take into account productivity or skill, these findings are not evident.

Last, as with any economic model, there exists the possibility of unpredictable market forces. For example, perhaps an increase in the number of public positions available will have little effect if the private sector grows by an unprecedented amount or if emigration to other nations’ medical systems becomes easier or more financially appealing.

## Discussion

Tanzania is a long way from having sufficient doctors to serve its population. According to a 2012 estimate of 0.31 doctors per 10,000 population, Tanzania has the seventh-lowest physician-to-population ratio of all nations (9). In addition, there is a dramatic difference in access between people living in rural *versus* urban areas. For example, the 2010 Tanzanian Demographic and Health Survey reported that out of all women who had given birth in urban areas in the last 5 years, 7.8% had received antenatal care from a doctor and 10.4% had been delivered by a doctor compared to 2.1 and 3.5%, respectively, for women in rural areas (41).

It is appropriate that Tanzanian universities are admitting more medical students in order to have more doctors to deliver the Tanzania Development Vision 2025 and beyond. In fact, our model suggests that Tanzania will have trained enough doctors to reach 1.4 practicing doctors per 10,000 population by 2025 – a remarkable achievement. The actual number of doctors trained may be even higher than the model predicts, since we assumed a steady rate of 1,580 students enrolling every year after 2015, the capacity of programs approved by the Tanzania Commission for Universities for the 2015/2016 academic year (10). New medical schools continue to open and existing schools continue to expand enrollment. Thus, the number of students enrolling in and graduating from medical schools between 2016 and 2025 may escalate well beyond the number used in the model.

The model demonstrates that there are massive losses in the deployment of those trained. We estimate that of those who will graduate medical school between 2016 and 2025, 6,400 will be lost to clinical practice, representing a loss of 50%. Even more serious, in rural areas the physician-to-population ratio will improve very little over the next decade and will only reach 0.55 by 2025. The model predicts that the ratio of doctors to population in urban areas will reach 2.6 per 10,000 population – five times higher than the ratio for rural areas.

We identified several key points in which those setting out to become doctors are lost to clinical practice. The first is before students graduate. There are no definitive figures for the numbers of students entering Tanzanian medical schools who do not graduate as doctors, but Leon et al. demonstrate low motivation on entering and leaving medical school (11). If the figure is as high as the 10% we estimate in the model, then 1,370 of the 13,700 students who enter medical school from 2011 to 2020 will never practice medicine. Strategies to improve graduation rates would include admission policies that require potential candidates to write statements of purpose or be assessed through a pre-admissions interview, two policies that are not currently implemented at Tanzanian medical schools (25, 42, 43). Although medical schools in Tanzania are some of the strongest in their region, they are often challenged by a lack of staff, equipment, and facilities while being overwhelmed by too many students (38, 44). Mshana and Pemba suggest that policies focused on improving the curriculum, especially competency-based curricula, as well as upgrading equipment, will assist educational institutions (37).

The second key LP is when a qualified doctor no longer practices clinically. This can occur at any point in their careers when they become unemployed or move to a non-clinical or administrative job. Some graduates never actually practice medicine, perhaps because they have lost motivation and sought another type of employment or because they were unable to find a job (11, 45). We estimated that 41% of all medical school graduates between 2016 and 2025 will be lost to clinical practice in Tanzania for these reasons by 2025. A major concern is the number of graduates who cannot find employment immediately after graduating (15). The inequity between graduating physicians and available clinical positions may be due to poor coordination between different ministries responsible for provisioning and funding these different sectors (46). We demonstrate that Tanzania could reach an acceptable physician-to-population ratio earlier by scaling up the employment of recent graduates in public positions. This is consistent with the suggestions of Sirili et al., who argue that large portions of doctors in Tanzania are lost in the transition from medical school to employment (15).

The third key LP concerns qualified doctors choosing to practice abroad. Indeed, 24–52% of Tanzanian-trained doctors worked abroad according to studies published in 2006 and 2008 (2, 47). Major reasons for poor job satisfaction and emigration include low pay, poor work opportunities and environment, and lack of access to training and advancement opportunities (48–50). We estimated that 1.6% of clinically active doctors leave the country each year, based on evidence of the number of Tanzanian-trained doctors working abroad. In the model, 9% of all medical school graduates between 2016 and 2025 will go overseas by 2025, demonstrating that this is not the same scale of problem as internal migration from clinical practice.

The fourth and fifth key LPs relate to choices that doctors make to work in urban rather than rural areas. We demonstrate that if this loss could be prevented, the rural ratio of doctors per 10,000 population could reach 1.5 per 10,000 by 2020. Researchers in Ethiopia, Rwanda, Ghana, and the United States have found that improvements in infrastructure (including equipment), supportive management, study leave, and pay can increase students’ willingness to accept a rural posting by 11-fold and maintain a 5-year retention of 90%, especially when benefits are packaged in bundles (30, 50–52). We explored the development of a rural fellowship for some medical students to encourage them to work in rural areas. Although we cannot predict the overall success of such an intervention, we are able to show the changes in numbers working in rural areas for different success rates.

This project has revealed areas for future research. Creating the model demonstrated to us the dearth of recent information available not only about the number of Tanzanian graduates but also about what happens to them after they graduate (53). Some efforts have been made to track health professionals in Tanzania by MUHAS, Sikika, and the MoHSW (35, 45, 53), but we could not find reliable information to populate many parameters in the model. The new human resources for health information system implemented by the MoHSW will help tracking of all cadres of health care workers (35), but its data must feed into human resource and education policy decisions to ensure its utility.

Another key area for future research is into determining the factors for physician employment. Discrete-choice experiments completed with Tanzania's clinical officers and outside of Tanzania prove that these investigations are fruitful (50, 51, 54). They should be replicated for physicians and recent medical school graduates in Tanzania.

## Conclusions

To our knowledge, this is the first model exclusively focusing on the education and employment of physicians in Tanzania. While the model highlights the need for more information on medical school graduates, it provides useful insights into the attrition of doctors trained in Tanzania. The model suggests that upwards of 56% of medical students enrolled between 2011 and 2020 will not be practicing medicine in 2025. In light of these findings, Tanzanian policy makers would be well served to increase the health system's capacity to absorb additional graduating students and improve physician working conditions, especially in rural areas. Without these efforts, the goals laid out in Tanzania's Development Vision 2025 may be jeopardized.

Tanzania is not alone in these challenges: 22 other nations in Africa have physician-to-population ratios under the minimum recommendation of one per 10,000 (9). Most of these nations are increasing enrollment at established schools and witnessing the development of new (largely private) medical schools (55). The pathways through medical education and graduate employment and the LPs that we describe are similar in these settings. Without targeted interventions, we anticipate that other African countries will face similar challenges in employing their increasing number of graduates. The dearth of data available for the model highlights the need to collect information that will inform the development of interventions to make the best use of this emerging physician workforce.

## Paper context

Tanzania is challenged with one of the world's lowest physician-to-population ratios. In response, universities have rapidly scaled up admissions. We built a model to track medical students and physicians from 1990 to 2025. The model predicts enormous attrition from clinical practice, especially from rural areas. Interventions such as increasing the number of public positions available to recent graduates and developing rural-focused training fellowships may avert this attrition and help Tanzania reach acceptable physician-to-population ratios nationwide.
